# Non-Diffractive Bessel Beams for Ultrafast Laser Scanning Platform and Proof-Of-Concept Side-Wall Polishing of Additively Manufactured Parts

**DOI:** 10.3390/mi11110974

**Published:** 2020-10-30

**Authors:** Huu Dat Nguyen, Xxx Sedao, Cyril Mauclair, Guillaume Bidron, Nicolas Faure, Enrique Moreno, Jean-Philippe Colombier, Razvan Stoian

**Affiliations:** 1 Laboratoire Hubert Curien, UMR 5516 CNRS, Institute of Optics Graduate School, Université de Lyon, Université Jean Monnet, 42000 Saint-Etienne, France; huu.dat.nguyen@univ-st-etienne.fr (H.D.N.); xxx.sedao@univ-st-etienne.fr (X.S.); nicolas.faure@univ-st-etienne.fr (N.F.); enrique@moreno.ws (E.M.); jean.philippe.colombier@univ-st-etienne.fr (J.-P.C.); razvan.stoian@univ-st-etienne.fr (R.S.); 2 GIE Manutech-USD, 42000 Saint-Etienne, France; guillaume.bidron@manutech-usd.fr

**Keywords:** Bessel beam, non-diffractive, self-healing, ultrafast laser, surface processing, additive manufacturing, selective laser melting, laser powder bed fusion, scanner

## Abstract

We report the potential use of non-diffractive Bessel beam for ultrafast laser processing in additive manufacturing environments, its integration into a fast scanning platform, and proof-of-concept side-wall polishing of stainless steel-based additively fabricated parts. We demonstrate two key advantages of the zeroth-order Bessel beam: the significantly long non-diffractive length for large tolerance of sample positioning and the unique self-reconstruction property for un-disrupted beam access, despite the obstruction of metallic powders in the additive manufacturing environment. The integration of Bessel beam scanning platform is constructed by finely adapting the Bessel beam into a Galvano scanner. The beam sustained its good profile within the scan field of 35 × 35 mm${}^{2}$. As a proof of concept, the platform showcases its advanced capacity by largely reducing the side-wall surface roughness of an additively as-fabricated workpiece from Ra 10 μm down to 1 μm. Therefore, the demonstrated Bessel–Scanner configuration possesses great potential for integrating in a hybrid additive manufacturing apparatus.

## 1. Introduction

Additive manufacturing (AM) is a cutting-edge technology that relies on builds-up of consecutive layer-by-layer parts for fabricating complex three-dimensional (3D) objects [1]. The technology enables the transfer of digital designs to direct production of near-net shaped objects. It thereby offers great enhancement over the degree of freedom regarding shape complexity when compared to traditional subtractive manufacturing [2,3]. In particular, laser powder bed fusion process (LPBF), also known as selective laser melting (SLM) is a well-established AM technique that uses an incoming laser beam as a thermal energy source to selectively melt, fuse metallic powders, and build up thin additive layers. The LPBF technique allows for manufacturing 3D parts with high complexity, dimensional control, and spatial resolution [4]. The technique can fabricate a wide variety of powder materials, including Fe-based [5], Al-based [6], Ti-based [7], Cu-based [8], Ni-based [9], and Co-based alloys [10]. However, despite the production of near-net shaped objects, the LPBF experiences some shortcomings concerning the surface roughness of as-fabricated parts, which relates to several physical phenomena: i.e., conduction heat transfer, balling effects, and tension gradient on the melt surface; and, process variables: i.e., morphology of powders, spot size, power, speed, and trajectory of the scanning beam [11].

The top surface roughness of the LPBF-fabricated parts can be strategically tuned with the process variables [11] and laser re-melting approach [12]. However, the removal of side-wall roughness requires a certain amount of post-processing treatments: i.e., computer numerical control (CNC) milling, grinding, chemical, electrochemical etching, and laser surface polishing [12,13]. The mechanical post-processing techniques tend to be limited for fabricated parts with a high degree of geometrical complexity, i.e., inaccessible internal surfaces. The chemical approaches are limited over a control of surface topography and amount of material removal [14]. Among the choices, the laser surface treatment offers great control over the surface-finished quality, high flexibility over complex internal surface parts, and high potential to integrate into the existing LPBF apparatus for simultaneous hybrid machining without the need of post-processing [13,15,16,17].

For surface processing using conventional continuous-wave (CW) and long pulsed lasers, the typical timescale of the pulses is much larger than the photon-electron-lattice energy coupling time. The primary mechanism of material removal is accompanied by strong heating, melting, and eventually vaporising with molten metal being ejected by a gas jet [18]. This process results in significant issues of recast/redeposited metal layers and melted debris [18]. Ultrafast pulsed lasers emerge as an efficient micro-processing technique which takes advantage of the ultrafast deposition of photon energy into the material under extreme non-equilibrium thermal conditions [19,20,21,22]. The use of laser pulses with sub-picosecond duration reduces significantly heated zone and mechanical damage to the workpiece and, therefore, promises a significant improvement of surface quality of additively fabricated parts [17].

The ultrafast laser processing on metallic materials is traditionally conducted with Gaussian beams [19,23,24,25,26,27]. However, using non-diffractive Bessel beam configurations could offer potential for further advancing the surface processing. Bessel beams represent a class of non- diffractive optical fields that ideally do not spread with propagation distance. Their formation is created by conical interference of rays upon propagation, which produces an invariant intensity profile in the axial direction along with a self-reconstruction property. The advantages of ultrafast laser Bessel beams had been effectively demonstrated for high aspect ratio structuring of transparent materials [28,29,30,31,32,33,34,35,36]. Even if they require transparent media to be formed, specific advantages can be expected when processing non-transparent/metallic materials, particularly LPBF-fabricated parts. The long diffraction-free length of the beam offers its advantage for large tolerance of sample positioning, while its self-reconstruction property enables the beam to be efficiently delivered to a targeting surface.

In this work, we report the potential use of the Bessel beam for ultrafast laser processing in additive manufacturing environment, its integration into a fast scanning platform, and proof-of-concept side-wall polishing of additively manufacture parts. The ultimate goal is to integrate the entire setup into a dual-beam hybrid manufacturing system [17]. The zeroth-order Bessel beam is generated while using an axicon and demagnified through a telescopic afocal arrangement. The resulted Bessel beam possesses a significantly long non-diffractive depth of focus, which gives it advantages over sample positioning during manufacturing processes. The self-reconstruction signature of the laser Bessel beam is experimentally studied under the obstruction of metallic microparticles and numerically illustrated. This property is advantageous for machining in the additive environment, where it is typically contaminated by inherent cloud of particles, which partially limits the access of laser beam. In the effort of upscaling the manufacturing process, the Bessel beam is coupled to a Gavalno scanner, enabling Bessel beam scanning in a large-scaled platform. As a proof of concept, the platform facilitates the Bessel beam advancement in side-wall polishing on the LPBF-fabricated workpiece, which is successfully demonstrated with the roughness improvement from Ra = 10 μm down to 1 μm.

## 2. Results and Discussion

### 2.1. Bessel Beam and Its Self-Healing Property under Particle Obstruction

An ideal zeroth-order Bessel beam is defined as the beam whose electric field (E) is explicitly described by the zeroth-order Bessel function of the first kind ($J_{0}$) [37,38]:

(1) $$E\left(r,\phi ,z\right)=A_{0}J_{0}\left(k_{r}r\right)e^{jk_{z}z}$$

where $A_{0}$ is the amplitude of the electric field; $k_{z}$ and $k_{r}$ are longitudinal and radial wavevectors, with $k=\sqrt{k_{z}^{2}+k_{r}^{2}}=2\pi /\lambda$ is the wavenumber in air at the wavelength λ; *z*, *r*, and ϕ are the longitudinal, radial, and azimuthal components, respectively.

The beam transversally appears in the form of a maximum intensity central lobe, surrounded by an infinite series of concentric rings with lower intensity. Its formation is considered to be a result of the interference of plane waves whose wavevectors belong to a conical surface. Experimental Bessel beams carry a definite amount of energy due to the finite extension of optical systems and use of practical beams.

In practice, the generation of Bessel beams can be realised by several methods: annular slit method [39], SLM based method [40,41], optical fibers [42,43], and axicons [28,29,30,44].

**Figure 1:**
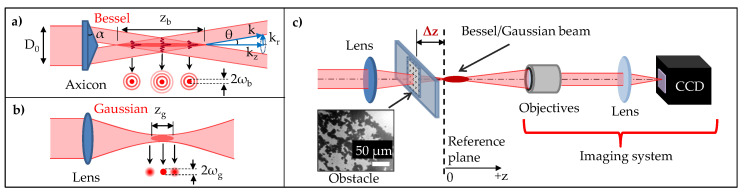
(**a**) Schematic of the non-diffractive Bessel beam generated by an axicon. The beam profile consists of a high intensity central core surrounded by a series of low intensity concentric lobes. The beam is defined by its half conical angle θ, depth of focus (Bessel length) $z_{b}$, and diameter of the central core 2$\omega_{b}$ at 1/$e^{2}$. (**b**) The Gaussian beam generated by a focusing lens, with a depth of focus $z_{g}$ = 2 × Rayleigh range, and a beam diameter 2$\omega_{g}$ at 1/$e^{2}$. (**c**) The setup for investigating propagation of the beams through obstruction by metallic particles immobilised on a glass slide (inset-microscope image). With Δz being the distance between the obstacle and the starting point of a beam focus (the reference plane).

In our work, a high quality axicon or conical lens is chosen as an efficient way for generating the so-called Bessel–Gaussian (BG) beam. When collimated Gaussian laser beams pass though an axicon with a refractive index n and a base angle α, they deviate with an angle θ=arcsin(nsin(α))−α to the optical axis (Figure 1a). The conical intersection of wavefronts upon propagating through the axicon creates an interference pattern which is characterised as the zeroth-order Bessel beam with the conical half-angle θ. The transverse profile of the beam comprises a bright central core that is surrounded by concentric rings (Figure 1a). The full width/diameter of the central lobe (2$\omega_{b}$) can be determined by the first zeros of the Bessel function [37]:

(2) $$2\omega_{b}=\frac{2\kappa }{k\sin(\theta )};\kappa \approx 2.405.$$

The beam central lobe has the highest intensity and it does not spread within the non-diffractive propagation (Bessel zone). The length of the Bessel zone $z_{b}$ can be approximated from the wavevectors of the Bessel beam propagating in the conical pattern [45]:

(3) $$z_{b}=\frac{D_{0}}{2}\frac{k}{k_{r}}=\frac{D_{0}}{2\tan(\theta )}$$

where $D_{0}$ is the diameter of the incident Gaussian beam. At any specific transverse plane within the Bessel zone, the energy density in the central lobe will originate from a corresponding ring in the initial Gaussian distribution that is refracted by the axicon on this area. The peak fluence of the central lobe ($F_{b}$) can be approximated by its energy $\left(2\omega_{b}/D_{0}\right)E_{in}$ distributed over the surface area $\pi \omega_{b}^{2}$ [29]:

(4) $$F_{b}=\frac{2\omega_{b}}{D_{0}}\frac{2E_{in}}{\pi \omega_{b}^{2}}=\frac{4E_{in}}{\pi D_{0}\omega_{b}}$$

where $E_{in}$ is the Gaussian input energy. The peak fluence can be expressed as a function of the half conical angle θ:

(5) $$F_{b}=\frac{2E_{in}}{\pi \omega_{b}z_{b}\tan\left(\theta \right)}$$

In comparison to the conventional Gaussian beam which is generated by a focusing lens (Figure 1b), the Bessel beam offers a significantly longer (typically one order of magnitude longer) depth of focus than the Gaussian counter part with a similar beam diameter (2$\omega_{g}∼2\omega_{b}$). In the Gaussian setup, the elongation of the depth of focus ($z_{g}$ = 2 × Rayleigh range) would require lenses with long focal length, and incident beams with small beam diameter. This classic configuration has a limited extent and unwantedly makes the focused beam having a large beam diameter, which, in turn, limit applications on processing objects with small features. In this matter, the Bessel configuration could ideally offer the beam with a long depth of focus, while maintaining a small beam diameter.

The Bessel self-reconstruction property has not been reported in the harsh and dusty AM context, despite it being demonstrated in various studies [46,47,48]. In order to demonstrate the interest of the unique Bessel self-reconstruction property for laser processing in the AM environment, the propagation of beams through an obstruction created by a cloud of metallic particles was investigated. The experiment was conducted by a beam-profiling setup as illustrated in Figure 1c. The metallic particles with the size ranging between 5 and 45 μm were immobilised on a glass slide and covered ∼50% of its transmissive window (Figure 1c inset). The obstruction resembles the SLM manufacturing environment in which the presence of suspending metallic particles is inevitable. The beam transverse profiles after passing through the particles-immobilised glass slide (obstacle) were collectively recorded by the imaging system, which consists of an objective 50x, a lens f-100 mm and a CCD camera. The obstacle was placed at different positions (Δz) with respect to a starting point (the reference plane) of the focused beams. The profiles of both Bessel and Gaussian beams were studied under the same obstructed conditions for a fair comparison.

**Figure 2:**
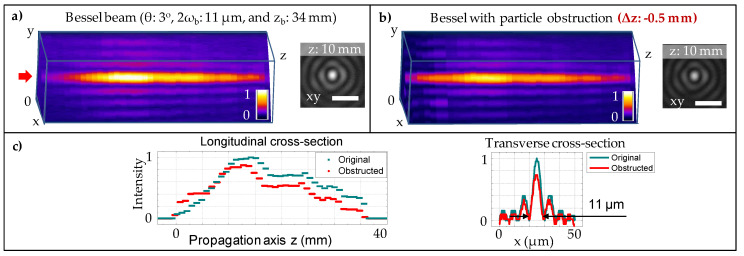
(**a**) Experimental analysis of the Bessel beam profile generated by an axicon and demagnified through a telescopic afocal arrangement. The beam possesses a long Bessel length $z_{b}$ = 34 mm, and a diameter of central core 2$\omega_{b}$ = 11 μm. (**b**) Evidence of seal-healing behaviour when the particle obstacle was placed at a distance Δz = −0.5 mm before the starting point of the Bessel focused region. The three-dimensional (3D) illustration was constructed by a series of transverse profiles which was individually captured at different position along the propagation axis z. A depicted transverse beam profile was shown at z: 10 mm. The scale bar: 20 μm. (**c**) Comparison of the original Bessel beam vs its obstructed profile: with longitudinal cross-section along the central lobe and transverse cross-section at z =10 mm. The red arrow indicates the laser propagation direction.

In the case of Bessel configuration, the Bessel beam was initially generated by focusing an incident pulsed laser beam (1 kHz, 200 fs, 800 nm) with a diameter of 4 mm through an axicon with $\alpha =5^{\circ }$. The generated beam was further demagnified through a telescopic afocal arrangement (lens1: 100 mm and lens2: 75 mm); giving a better adjustment of the Bessel beam parameters, i.e., a half conical angle θ = $3^{\circ }$, a central lobe 2$\omega_{b}$ = 11 μm, and a Bessel length $z_{b}$ = 34 mm (Figure 2a). The choice of axicon and lenses was made for the purpose of producing the Bessel beam with the Bessel length staying within our measureable tool (<50 mm), and a beam diameter large enough for comparison with a Gaussian counter part. Transverse profiles of the laser beams were individually captured at different positions with respect to the reference plane. Therefore, it is possible to construct 3D images of the beam profile by stacking those individual transverse images together. The stacked profile of Bessel beam shows a bright central lobe and surrounding rings. The central lobe diameter seems to exhibit a negligibly small variation (below 4 %) along the Bessel length, which could be attributed to a small divergence of the incident Gaussian beam as well as the imperfect tip of the conical lens [49]. The Bessel beam quality can be improved while using a spatial filter and/or using reflective axicon elements.

**Figure 3:**
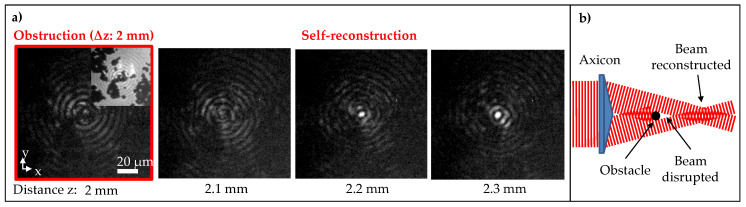
(**a**) Transverse profiles of the Bessel beam (θ = $3^{\circ }$, 2$\omega_{b}$ = 11 μm and $z_{b}$ = 34 mm) after obstruction of particles placed at Δz = 2 mm. Evidence of the obstruction occurs at the obstacle plane z = 2 mm, in which the inset shows the presence of the particles when additional background illumination was used for better visualisation. The beam started to reconstruct itself after 0.2 mm with respect to the obstacle plane. (**b**) Simplified schematic of the Bessel self-reconstruction mechanism. The beam disrupted shadow length can be geometrically approximated, depending on the beam conical angle, size, and location of the obstacle.

The Bessel self-healing behaviour is evidently confirmed by the reconstructed beam profile presented in Figure 2b, in comparison with the original profile without obstacle in Figure 2a. It is noted that the experiment was focused on the self-reconstruction of the beam central lobe. In the particular case, when the obstacle was placed at Δz = −0.5 mm before the reference plane, the Bessel beam did not suffer from the obstruction. Figure 2c shows longitudinal and transverse cross-section of the original Bessel beam vs its self-reconstructed profile. The self-reconstructed beam sustained a good profile, except a small decrease of <10 % in its peak intensity along the central region (Figure 2c). A closer look to the evolution of the self-reconstruction (Δz = 2 mm) is additionally illustrated in Figure 3a, where the obstruction clearly occurred at the position 2 mm (Inset shows the image with an additional background light for better visualisation). The beam started to reconstruct itself at the position 2.2 mm, which is just 0.2 mm after the obstruction. The beam reconstruction mechanism is described in a simplified schematic (Figure 3b), where the length of the beam disrupted shadow is approximately the distance to which the beam starts reconstructing. The length can be geometrically estimated, depending on the beam conical angle, size, and location of the obstacle [37]. Particularly, in the presence of 4–45 μm particles, the shadow length is approximated within a range between 0.2 mm and 1 mm. Similar self-reconstructed results were observed when the obstacle was placed at Δz = −6, −3, −2, −1, −0.5, 0, 0.5, 1, 2, 3, and 6 mm planes with respect to the reference plane of the Bessel beam (data not shown for the sake of brevity).

**Figure 4:**
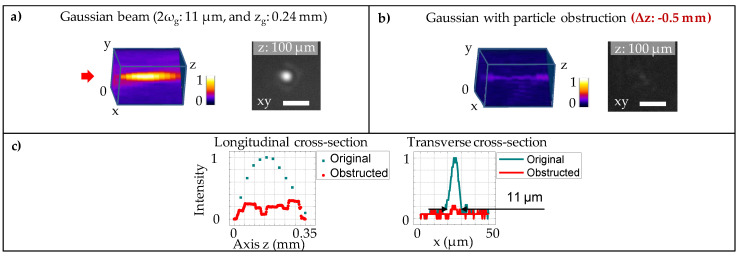
(**a**) Experimental analysis of the Gaussian beam profile generated by a focusing objective 4x 0.1NA. The beam has a short depth of focus $z_{g}$ = 0.24 mm and a beam diameter 2$\omega_{g}$ = 11 μm. (**b**) Evidence of the beam distortion in the presence of obstacle placed at Δz = −0.5 mm before the starting point of the Gaussian focused region. The 3D illustration image was obtained with a similar procedure as presented for the Bessel beam. The transverse beam image was shown at the position z = 100 μm. The scale bar: 20 μm. (**c**) Comparison of the original Gaussian beam and its distorted profile: with longitudinal cross-section at the beam central and transverse cross-section at z = 100 μm.

For comparison, the Gaussian beam was separately evaluated in the same particle obstruction, using an objective 4x, NA 0.1, which gives the focused beam diameter 2$\omega_{g}$ = 11 μm relatively similar to its theoretical calculation, and comparable with the central lobe diameter of the presented Bessel beam. A small aberration (ring shadow) can be seen in the Gaussian beam profile (Figure 4a). This is a common effect that is caused by the finite aperture of the incident beam. The Gaussian beam is characterised with a short depth of focus/confocal region $z_{g}$ = 0.24 mm, measured where the beam intensity drops by a half. This measured value is slightly smaller that its theoretical calculation, which is attributed to the laser beam quality factor M${}^{2}\approx$ 1.33. The short confocal region of the Gaussian beam evidently exhibits its limit over the long non-diffractive Bessel length $z_{b}$ = 34 mm. Concerning the beam profile after passing through the obstacle (particularly placed at Δz = −0.5 mm), the result shows a distorted form of the beam (Figure 4b). The distorted beam experienced a large drop of its intensity down to ∼20% (Figure 4c). Similar distortions were recorded when the obstacle was placed at Δz = −6, −1, −0.5, and 0 mm with respect to the reference plane of the Gaussian beam (data not shown for the sake of brevity).

**Figure A1:**
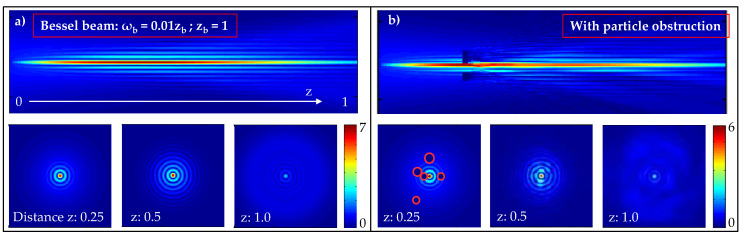
(**a**) Simulated profiles of a zeroth-order Bessel beam with a normalised Bessel length $z_{b}$ = 1, and a central core diameter 2$\omega_{b}$ = 0.01$z_{b}$. The beam transverse profile consists of a bright central core and surrounding circular lobes. (**b**) Simulated profiles of the Bessel beam in the presence of particle obstruction. The beam reconstructs itself after the obstruction at the plane z = 0.25. The reconstructed beam suffers a small decrease in its peak intensity. The particles are with a diameter of ∼0.015$z_{b}$.

**Figure A2:**
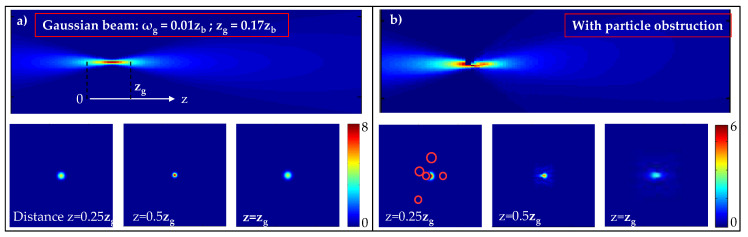
(**a**) Simulated profiles of a Gaussian beam with a normalised depth of focus $z_{g}$ = 0.17$z_{b}$ and a beam diameter 2$\omega_{b}$ = 0.01$z_{b}$. (**b**) Simulated profiles of the Gaussian beam under the same particle obstruction condition at the plane z = 0.25$z_{g}$. The beam distorts and loses its intensity.

The experimental results of both Bessel and Gaussian beams were in a good agreement with numerical simulation (see Appendix: Figure A1 and Figure A2). The successfully demonstrated advantages of the Bessel beam properties open great potential for the Bessel configuration to be applied in large-scale processing.

### 2.2. Bessel Beam Scanning Platform

**Figure 5:**
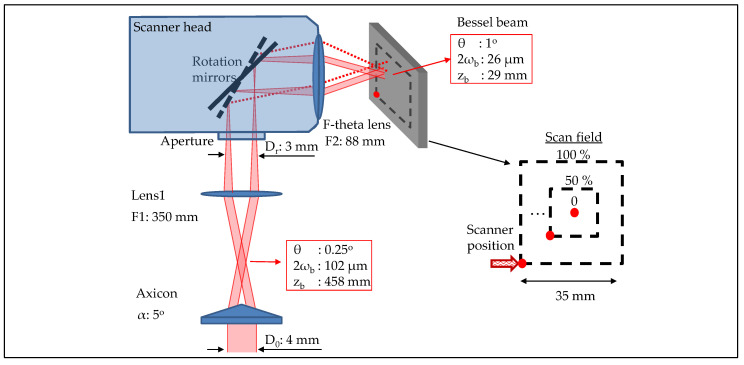
Simplified schematic of setup for coupling Bessel beam with a Galvano scanner—Bessel scanning platform. The Bessel beam is formed by an axicon ($\alpha =0.5^{\circ })$, and imaged through a telescopic afocal arrangement (lens1 F1 = 350 mm and F-theta lens F2 = 88 mm). The platform provides the Bessel beam with a half conical angle $\theta =1^{\circ }$, a diameter of central core 2$\omega_{b}$ = 26 μm, and a long non-diffractive length $z_{b}$ = 29 mm. Profile of the beam is examined when the scanner moves from centre to corner (red point) of the scan field with the full extent 100% equivalent to 35 mm.

The previous demonstration of the Bessel beam for its beneficial diffraction-free depth of focus and self-reconstruction property, along with its efficiency for surface ablation [50], have drawn the attention for its adaptation into a large-scale laser processing platform, i.e., a scanner. Despite the Bessel beam-scanner integration being demonstrated for microscopy [51,52], coupling of a high power Bessel beam to a large-scale scanner for machining has not yet been done. In this approach, the Bessel beam scanning platform was realised by combining an axicon with a telescopic afocal arrangement in which one of the lenses, i.e., lens2 was a f-theta lens of a Galvano scanner (Figure 5). The setup was additionally equipped with a telescope which helps tuning the incident beam size, and accordingly adjusting the Bessel length. The particular axicon was used with a base angle α = $5^{\circ }$, in combination with a 4 mm diameter incident beam, resulting in the initial Bessel beam with a half conical angle θ = $0.25^{\circ }$, a central core diameter 2$\omega_{b}$ = 102 μm, and a Bessel length $z_{b}$ = 458 mm. After demagnification from the telescopic system: the lens1 F1 = 350 mm and the f-theta lens F2 = 88 mm, the final Bessel beam was characterised with θ = $1^{\circ }$, 2$\omega_{b}$ = 26 μm, and $z_{b}$ = 29 mm. It is noted that the choice of the axicon and lenses was taken when considering the production of a beam central lobe diameter comparable with its Gaussian counter part, and a well fit of the intermediate incoming Bessel ring to the entrance aperture (3 mm diameter) of the scanner head. The size of the intermediate Bessel ring is critical to adapt in most of the cases, which requires a combination of an axicon to telescopic afocal systems, particularly when the beam encounters a small aperture.

The beam quality is transversally characterised within the scan field of 35 × 35 mm${}^{2}$) in order to examine the stability of the Bessel beam formation through the scanner. The scanner head allows for the beam to move to different zones from the centre to the furthest corner of the scan field (Figure 5 red point) where its profile is captured by the imaging beam profiler.

**Figure 6:**
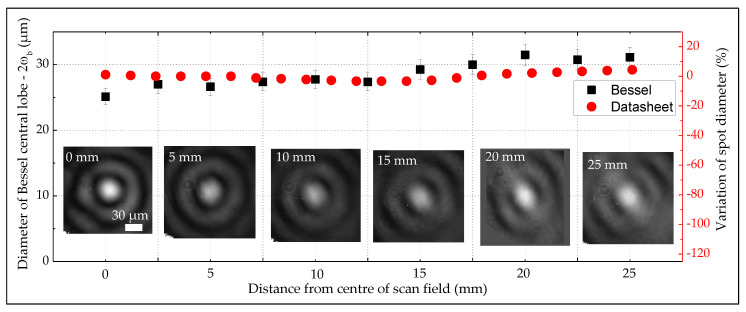
Profile of the Bessel beam and diameter of its central core 2$\omega_{b}$ as a function of the distance from the centre to corner of the scan field. The experimental data (Square-symbol graph) are well agreed with datasheet (Circle-symbol graph) of the f-theta lens. Qualitatively, the Bessel beam sustains its good form over the full extent of the scan field.

The resulted profile of the Bessel beam through the scanner, along with its central lobe diameter are illustrated as a function of the distance from the centre to corner of the scan field (Figure 6). Quantitatively, the beam appears to have a small variation in its lobe diameter along the field extent (Figure 6 Square-symbol graph). This is in a good agreement with values from the datasheet provided for a classic Gaussian configuration (Figure 6 Circle-symbol graph). In the datasheet graph, the diameter variation was adapted to the percentage unit, since its original data were provided for a different beam size. Concerning the beam spot distribution or the beam symmetry over the scan field, little degradation of the profile was observed at the furthest extent (25 mm from the scan field centre). The slight shape distortion of the beam is related to the telecentricity of the F-theta lens, which similarly occurs to the Gaussian configuration. The results were well in line with another independent experiment on a Gaussian beam profile while using the same f-theta lens conducted on the same scanner. Bessel and Gaussian beams both resulted in the same propagation axis, regardless of the position of the beams within the scan field.

It is worth noting that the given Bessel beam has the central lobe diameter (26 μm) comparable with a Gaussian beam diameter (27 μm) while using the same f-theta lens, whereas its depth of focus is proven to be >1 order of magnitude longer than the Gaussian counter part (29 mm as compared to 2 mm). By optimising the choice of the axicons and lenses, the platform can be further adjusted in order to produce the beam with a smaller diameter while maintaining its beneficial long depth of focus. There have been reports of taper-like shapes being produced during ultrafast laser processing, which limits the surface enhancement of the processed material. Therefore, the smaller-diameter beam is desirable for minimising its taper effect. While this is challenging for a classic Gaussian beam setup, the Bessel beam configuration can come in as a better alternative.

### 2.3. Side-Wall Polishing with Bessel Beam

**Figure 7:**
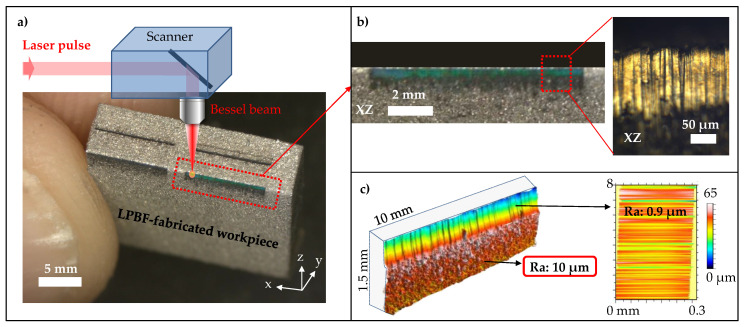
(**a**) Illustration of ultrafast laser side-wall polishing on a LPBF-fabricated workpiece, using the Bessel beam scanning platform with laser conditions: 2 kHz, 100 fs and 1 J/cm${}^{2}$. (**b**) A zoom in the laser-polished part, showing evidence of visually reflective surface with microscope image. The blue colour (red and green not shown) of the laser-polished region was separated from white light illumination due to reflection grating by sub-micro grooves which were formed under raster scanning of the laser beam. (**c**) Roughness measurement of the polished part, demonstrating a reduction of roughness Ra from 10 μm down to ∼1 μm. The colour bar represents topographical heights within the measured area.

As a proof of concept, the Bessel beam scanning platform was further used in order to showcase the Bessel beam advanced capacity for side-wall polishing on an additively as-fabricated workpiece (Figure 7). The experiment was performed with the laser Legend Elite from Coherent Inc, using optimised parameters: linear polarisation, repetition rate 2 kHz, pulse duration 100 fs, and peak fluence ∼1 J/cm${}^{2}$. These optimised laser conditions were chosen from [50], towards producing optimal ablation efficiency and effective roughness improvement of the ablated surface.

The dynamic ablation was processed on the sample edge by setting the scanner to move in raster scanning trajectories (Figure 7a). The laser path is translated in the x-axis direction, and consecutively shifted in the y-axis direction. The pulse-to-pulse overlap in x-axis depends on the scanning speed, the beam spot size, and repetition rate of the laser, while the y-axis overlap depends on the spacing shift between the consecutive scanning lines. In this demonstration, the overlaps in x-axis and y-axis were kept the same at 97%, towards the optimisation of the laser-polishing quality. It is noted that the laser translation was started from a position outside of the sample edge, and then y-shifted toward the sample bulk. After 20 iterative scans, the ablated and polished part at the edge of the LPBF-fabricated sample was qualitatively characterised by a microscope, showing evidence of visually reflective surface (Figure 7b). The polished region appears to split the white light illumination into red green blue (RGB) color (the blue was shown in the image). It is a result of the reflection grating by sub-micro grooves which were formed under raster scanning of the laser beam. For a quantitative characterisation, the laser-polished part is further measured for its side-wall roughness (Ra). The result reveals a roughness reduction of the LPBF-fabricated sample from its original side-wall roughness Ra of 10 μm to Ra of 1 μm (Figure 7c).

It can be noticed that the 0.3 mm polished depth was achieved (Figure 7c), but it can be extended following longer scanning time/cycles. However, achieving a long-depth polishing is not the scope of the paper. It is also pertinent to mention that the Rayleigh length of the Gaussian beam using the same F-theta lens is 1.3 mm, which is above 0.3 mm. Thereby, the polishing on 0.3 mm depth is also possible with the ex-situ Gaussian configuration. However, in the harsh AM environment, the Gaussian beam is much more sensitive to obstacles (inherent suspending particles), whereas the Bessel beam can self-reconstruct.

The Bessel beam scanning platform effectively demonstrated its proof-of-concept side-wall polishing and additionally promises its potential for re-dimensioning over-sized AM parts. It lays a foundation for the ultimate use of this beam engineering solution in a hybrid AM machine [16]. The hybrid AM machining is an inline process in which each AM layer can be immediately treated, prior to the next AM layer to be built, with a contour of ultrafast Bessel beam scanning. It allows for direct dimension controlling and polishing of the current AM layer’s sidewall. Therefore, the resulting AM parts do not require additional surface post-processing steps.

## 3. Materials and Methods

The work has been performed with the main optic components: i.e., the axicons (α = $0.5^{\circ }$ and $5^{\circ }$), lenses (F = 75 mm, 100 mm, and 350 mm) brought from Eksma Optics. The Objectives were used including: Olympus PLN 4x NA 0.1 and Mitutoyo Plan Apo 50x NA 0.42. The CCD camera was Thorlabs DCU224M 1280 × 1024 resolution. The Galvano scanner was intelliSCAN from SCANLAB, along with the f-theta lens f-88 mm bought from Jenoptik.

Metallic powders were the type 630 stainless steel or more commonly known as 17-4PH, with the microparticle size within a range of 4 and 45 μm. In preparation of the particle-immobilised obstacle, the particles were initially dissolved in distilled water, whose solution was then cast on top of a 1 mm thick glass slide. The particle-cast glass slide was additionally put in sandwich with a 500 μm thin glass film, and eventually dried out in ambient air.

For qualitative observation, the additively fabricated samples were characterised in a reflected-light Zeiss microscope. Their surface roughness was quantitatively measured by a chromatic confocal microscope (CL1 MG 201, STIL, Vaux-le-Pénil, France), and then analysed in MountainsMap software from Digital Surf. The surface roughness was quantified with Ra term, which is the arithmetic average value of peaks and valleys in a series of individual measurements.The value was recorded within a designated evaluation length (≈10 mm) and width (≈0.3 mm).

## 4. Conclusions

The work has illustrated the potential use of the Bessel beam for ultrafast laser processing in additive manufacturing environments, its integration into a Gavalno scanning system, and proof-of-concept side-wall polishing of additively fabricated parts. The Bessel beam was generated while using an axicon and a telescopic afocal system, giving a significantly long non-diffractive distance (34 mm). The self-reconstruction signature of the laser Bessel beam was rigorously clarified under obstruction of metallic microparticles. The unique properties give the beam great advantages for industrial machining, particularly in the additive manufacturing environment where the obstruction of suspending particles usually occurs. For upscaling the manufacturing process, the Bessel beam was successfully adapted to a Gavalno scanner. As a proof of concept, the realisation of a large scale Bessel beam processing platform facilitates the Bessel advancement in side-wall polishing on the additively fabricated material, with roughness improvement from Ra = 10 μm down to 1 μm. Therefore, the demonstrated Bessel–Scanner configuration proposes a handy approach for integrating in a hybrid processing machine.

## Abbreviations

The following abbreviations are used in this manuscript:

AM	Additive manufacturing
3D	3-dimensional
LPBF	Laser powder bed fusion
SLM	Selective laser melting
CNC	Computer numerical control
CW	Continuous-wave
BG	Bessel-Gaussian

## Appendix A

In order to numerically illustrate behaviour of the beams under the obstruction condition, the beam propagation was simulated by a Fresnel model [53]. The Fresnel propagation code was used to calculate the irradiation pattern over the focal volume, within the paraxial approximation and neglecting spectral effects. Figure A1 shows simulated profiles of (a) a zeroth-order non-diffractive Bessel beam and (b) its self-reconstruction under the obstruction of particles. The beam has a normalised Bessel length $z_{b}$ = 1, and a central core diameter 2$\omega_{b}$ = 0.01$z_{b}$. The beam encounters the obstruction of the particles at the plane z = 0.25, and reconstructs itself after a certain distance, although it suffers a small decrease in the peak intensity. A clearer evidence of the beam reconstruction progress was observed when comparing its transverse profiles depicted at the same planes z = 0.25, 0.5 and 1.0 before and after placing the particles. The spherical particles which have a diameter of ∼0.015$z_{b}$ are spread around the beam central region.

Figure A2 shows simulated profiles of (a) a Gaussian beam and (b) its propagation under the same particle obstruction condition. The beam has a normalised depth of focus $z_{g}$ = 0.17$z_{b}$ and a beam diameter 2$\omega_{b}$ = 0.01$z_{b}$. The beam distortion and its intensity loss were observed when comparing its profile before and after the particles are in place. Transverse profiles were depicted at the planes z = 0.25$z_{g}$, 0.5$z_{g}$, and 1.0$z_{g}$, for a clearer indication of the disrupted beam propagation.
